# Feasibility and safety of focused ultrasound-enabled liquid biopsy in the brain of a porcine model

**DOI:** 10.1038/s41598-020-64440-3

**Published:** 2020-05-04

**Authors:** Christopher Pham Pacia, Lifei Zhu, Yaoheng Yang, Yimei Yue, Arash Nazeri, H. Michael Gach, Michael R. Talcott, Eric C. Leuthardt, Hong Chen

**Affiliations:** 1 Department of Biomedical Engineering, Washington University in St. Louis, Saint Louis, MO 63130 USA; 2 Mallinckrodt Institute of Radiology, Washington University School of Medicine, Saint Louis, MO 63110 USA; 3 Department of Radiation Oncology, Washington University School of Medicine, Saint Louis, MO 63108 USA; 4 Division of Comparative Medicine, Washington University School of Medicine, Saint Louis, MO 63110 USA; 5 Department of Neurosurgery, Washington University School of Medicine, Saint Louis, MO 63110 USA; 6 Department of Neuroscience, Washington University School of Medicine, Saint Louis, MO 63110 USA; 7 Center for Innovation in Neuroscience and Technology, Washington University School of Medicine, Saint Louis, MO 63110 USA

**Keywords:** Diagnostic markers, Biomedical engineering

## Abstract

Although blood-based liquid biopsy is a promising noninvasive technique to acquire a comprehensive molecular tumor profile by detecting cancer-specific biomarkers (e.g. DNA, RNA, and proteins), there has been limited progress for brain tumor application partially because the low permeability of the blood-brain barrier (BBB) hinders the release of tumor biomarkers. We previously demonstrated focused ultrasound-enabled liquid biopsy (FUS-LBx) that uses FUS to increase BBB permeability in murine glioblastoma models and thus enhance the release of tumor-specific biomarkers into the bloodstream. The objective of this study was to evaluate the feasibility and safety of FUS-LBx in the normal brain tissue of a porcine model. Increased BBB permeability was confirmed by the significant increase (p = 0.0053) in K^trans^ (the transfer coefficient from blood to brain extravascular extracellular space) when comparing the FUS-sonicated brain area with the contralateral non-sonicated area. Meanwhile, there was a significant increase in the blood concentrations of glial fibrillary acidic protein (GFAP, p = 0.0074) and myelin basic protein (MBP, p = 0.0039) after FUS sonication as compared with before FUS. There was no detectable tissue damage by T_2_^*^-weighted MRI and histological analysis. Findings from this study suggest that FUS-LBx is a promising technique for noninvasive and localized diagnosis of the molecular profiles of brain diseases with the potential to translate to the clinic.

## Introduction

Tissue biopsy has been used to characterize and track the tumor molecular landscape. However, tissue biopsy for brain tumor diagnosis requires invasive surgical procedures, which carry a 5–7% risk of major morbidity^1^. It may not be possible at all to perform this procedure on medically inoperable patients or patients with tumors in surgically inaccessible locations. Repeated tissue biopsies to assess treatment response and cancer recurrence are often not feasible given the increased risk for complications and morbidity. These challenges limit the timely diagnosis and selection of treatment options, hinder a better understanding of the disease, and impair the development of effective treatment approaches.

Liquid biopsy (LBx), which refers to the detection of tumor-derived components in body fluids (e.g., blood, urine, saliva, ascitic fluid, cerebrospinal fluid, etc.), has been gaining enormous attention in both medical research and clinical applications^2,3^. Various substances from liquid biopsies have been found to be closely related to the stage of a tumor and might serve as biomarkers for cancer diagnosis and prognosis, such as circulating tumor cells, circulating tumor DNAs, RNAs, extracellular vesicles, and a series of cancer-related proteins^4^. Blood-based LBx enables physicians to noninvasively interrogate the dynamic evolution of a tumor and monitor a patient’s response to therapies through a simple blood test. Blood-based LBx-guided personalized therapy has already entered clinical practice in the management of several cancers. An important example of blood-based LBx clinical utility is the assessment of epidermal growth factor receptor (EGFR) mutations in circulating tumor DNA to guide the use of EGFR tyrosine kinase inhibitors in patients with advanced-stage non-small-cell lung carcinoma^5,6^. However, extending blood-based LBx to brain cancer remains challenging^2^. Brain tumor blood-based LBx not only faces the challenge of establishing sensitive and reliable biomarker detection methods but also the unique challenge from the blood-brain barrier (BBB) that hinders the release of tumor biomarkers into the blood circulation^1^. A number of publications have demonstrated the ability to detect circulating brain tumor biomarkers in patients with brain cancer. Yet, brain tumor-derived biomarkers are generally detected at low abundance and in a limited number of patients, which makes analysis difficult in routine clinical practice^2,7–9^. With current biomarker detection techniques, circulating tumor DNA is detectable in >75% of patients with advanced pancreatic, ovarian, colorectal bladder, melanoma, and head and neck cancer, but only in <10% of glioma patients^2^.

Focused ultrasound (FUS) in combination with microbubbles has been established as a noninvasive BBB disruption technique for drug delivery (FUS-BBBD)^10^. Microbubbles, which are ultrasound contrast agents used in the clinic for ultrasound imaging, are intravenously injected into the blood circulation. FUS generated by an extracorporeal ultrasound transducer can penetrate through the skull and focus the ultrasound energy at a targeted brain location. When microbubbles pass through the FUS target brain region, the ultrasound waves induce microbubble cavitation (i.e., the expansion, contraction, and collapse of microbubbles in an acoustic field). The cavitating microbubbles localize and amplify the FUS acoustic energy and induce mechanical effects on the blood vessel wall^11^. Using optimized treatment parameters, FUS can transiently disrupt the BBB and increase its permeability without causing vascular damage. Successful applications of this technique have been demonstrated in not only various small animal models but also large animal models, such as nonhuman primates^12–15^, sheep^16^, and pigs^17–19^. Clinical trials are currently ongoing to evaluate the feasibility and safety of FUS-BBBD for brain drug delivery using magnetic resonance imaging (MRI)-guided FUS^20–22^. We proposed that FUS-induced BBB disruption enables a “two-way transfer” between the brain and blood circulation and introduced the FUS-enabled liquid biopsy technique (FUS-LBx)^23^.

Although the use of ultrasound to amplify biomarker signals in the blood was proposed in 2009^24^, most previous studies used high-intensity focused ultrasound (HIFU) to induce permanent mechanical or thermal disruption of tumors outside the brain to liberate biomarkers from tumor cells^25–27^. Following the initial introduction of the ultrasound-mediated biomarker amplification concept by D’Souza *et al*. in 2009, several *in vitro* studies were reported over the next few years^28–31^. These studies showed that ultrasound combined with microbubble-induced sonoporation could liberate various cellular contents into the extracellular space, such as enhanced green fluorescence protein^28^, mammaglobin mRNA^28^, micro-RNA 21^29^, cancer antigens 125 and 19–9^30^, and small molecule calcein^31^.

It was only after 2016 that *in vivo* studies on ultrasound-mediated tumor biomarker release started to be reported^25–27^. Chevillet *et al*. used pulsed HIFU to induce histotripsy (i.e., a technique for mechanical tissue fractionation) in a rat model of prostate cancer, and enhanced the release of cell-free tumor microRNA into the blood circulation^25^. Paproski *et al*. performed the experiment using a chicken embryo tumor model and demonstrated the feasibility of amplifying the release of extracellular vesicles using the mechanical damaging effect induced by HIFU in combination with phase-changing nanodroplets^26^. Souza *et al*. found significant increases in two protein biomarkers in the plasma of patients treated by HIFU thermal ablation of uterine fibroids^27^. All these previous studies used HIFU to induce permanent mechanical or thermal disruption of the tumor to enhance the release of tumor biomarkers from the tumor cells. The tissue-damaging effect limits the application of these techniques as diagnostic tools in a sensitive organ, such as the brain, and none of these techniques could resolve the BBB challenge inherent to brain tumors.

FUS-LBx is different from all previously reported strategies. Instead of using HIFU to disrupt the tumor tissue, FUS-LBx combines low-intensity pulsed FUS with microbubbles to overcome the unique challenge that the BBB poses on the efficient passage of tumor biomarkers from the brain into the peripheral circulation. We demonstrated the feasibility of FUS-LBx in murine glioblastoma tumor models^23^. In that study, we performed FUS-LBx after intracerebral implantation of glioma cells expressing the enhanced green fluorescent protein. The levels of green fluorescent protein mRNA in FUS-sonicated mice were 1,500–4,800 fold higher than those in control mice without FUS sonication. To demonstrate the clinical translation potential of FUS-LBx, large animal models are required because a small animal model cannot represent the technical challenge of FUS delivery through the thick human skull and biomarkers released by FUS-LBx will be far more diluted in humans and large animals than in mice. In the current study, we used pigs as the large animal model for demonstrating the feasibility of FUS-LBx. The pig model was selected because its similarity in blood volume/body weight, skull thickness, and brain morphology to humans^32–34^ and it has less ethical concerns compared with the primate model.

The objective of this study was to evaluate the feasibility and safety of FUS-LBx in a pig model to provide data that support the clinical translation of this technique. As healthy pigs without tumors were used, we could not evaluate the release of tumor-specific biomarkers. Instead, we selected brain-specific biomarkers as a proof of concept to demonstrate the feasibility and sensitivity of FUS-LBx. Specifically, glial fibrillary acidic protein (GFAP) and myelin basic protein (MBP) were selected to represent brain-specific biomarkers. These two biomarkers were selected for two reasons: they have been used as brain-specific biomarkers^35,36^, and protein biomarkers can be detected in the plasma using well-established enzyme-linked immunosorbent assays (ELISA).

## Methods

### Animal preparation

All animal procedures were reviewed and approved by the Institutional Animal Care and Use Committee at Washington University in St. Louis in accordance with the Guide for the Care and Use of Laboratory Animals and the Animal Welfare Act. A total of 16 pigs (age: around 4 weeks old; sex: male) were used in this study. These pigs were divided into two groups. The first group consisted of 8 pigs that were used to optimize the design of a customized MRI-guided FUS (MRgFUS) system for BBB opening in pigs and establish the standard operation procedure (SOP) for the FUS sonication of the pig brain. The second group consisted of 8 pigs that were used to evaluate the FUS-LBx technique using the optimized MRgFUS system and the established SOP. This Methods section describes the established SOP.

Pigs were sedated with an intramuscular injection of ketamine (2 mg/kg), xylazine (2 mg/kg) and telazol (4 mg/kg), intubated, and maintained under general anesthesia using isoflurane and positive pressure ventilation. The hair on the pig head was removed using depilatory cream (Nair, Church & Dwight Co., Princeton, NJ) to ensure optimal acoustic coupling. A catheter was placed in the ear vein for microbubble and MRI contrast agent injections. A fiber-optic pulse oximeter (Nonin 7500FO, Plymouth, MN) was used to monitor the blood oxygen level and pulse rate during the procedure. The animal body temperature was monitored and maintained with heated blankets.

### Customized MRgFUS device

**Figure 1:**
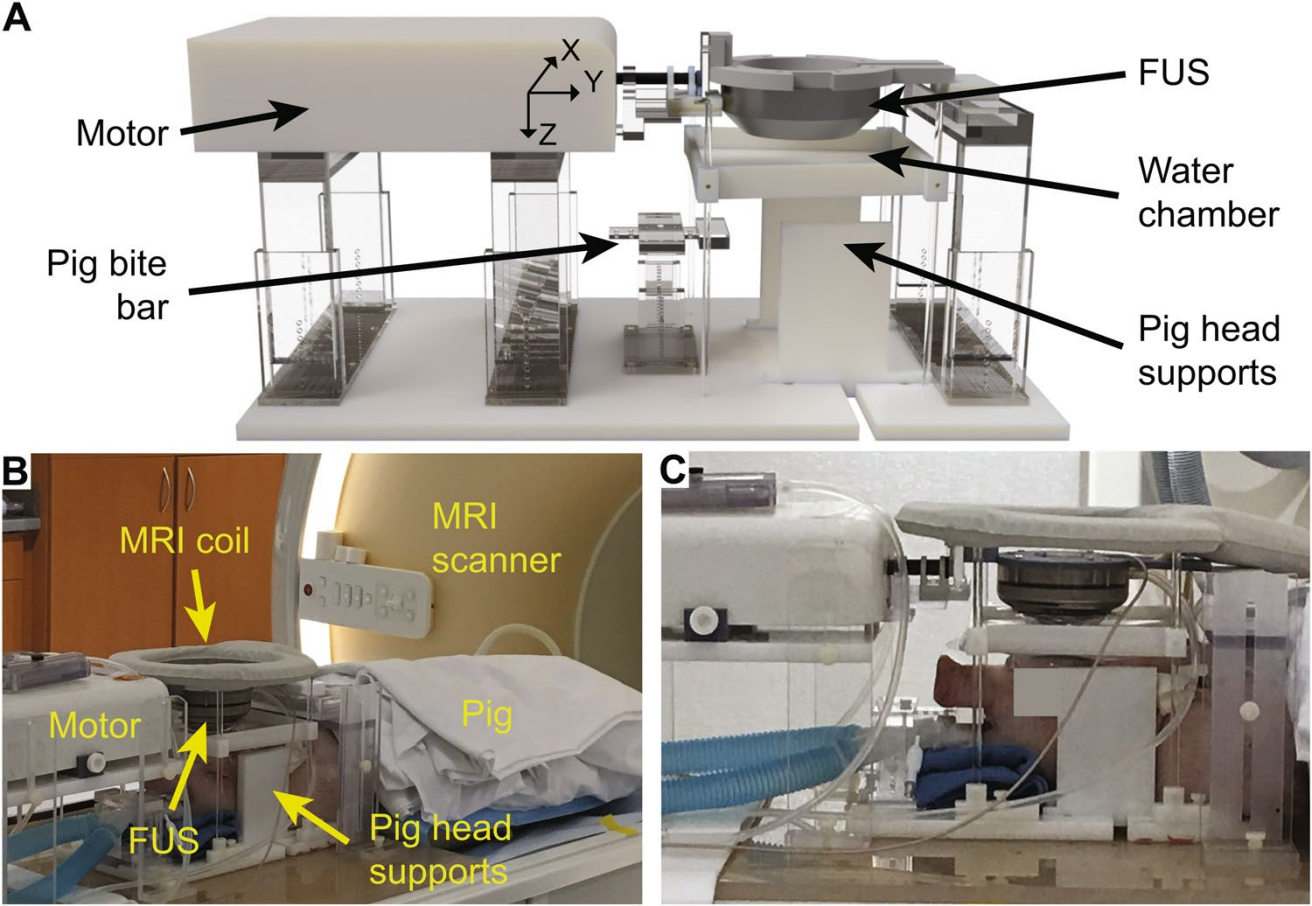
Customized MRgFUS hardware for BBB opening in pigs. (**A**) 3D rendering of the FUS system. An MRI-compatible motor moved the FUS transducer to target a specific brain area. The pig head was fixed and stabilized by a bite bar and two side-supports. The transducer was coupled with the pig head through a water chamber. **(B)** Picture of the MRgFUS system used during the pig study. **(C)** Close-up view of the pig head along with the FUS transducer, motor, and MRI coil.

An MRgFUS device was developed for the BBB opening in pigs. The major hardware components of the MRgFUS system are shown in Fig. 1. The FUS system was developed by modifying a commercial MRI-compatible FUS system (Image Guided Therapy, Pessac, France) that was originally designed for small animal applications by replacing the FUS transducer and changing the design of the animal support. The FUS transducer (Imasonics, Voray sur l’Ognon, France) used for the pig study was a 15-element annular array with a center frequency of 650 kHz, an aperture of 6.5 cm, and a radius of curvature of 6.5 cm. The transducer was spherically shaped with a hole in the center for integration with a single-element transducer with a center frequency of 650 kHz for passive cavitation detection (PCD). The PCD sensor had a center frequency of 650 kHz and a −6 dB bandwidth of 260 kHz. The transducer was driven by an RF generator (Image Guided Therapy, Pessac, France). The annular array design allowed the FUS transducer to electronically steer the focus in the axial direction of the transducer (Z-axis). The transducer was connected to an MRI-compatible piezoelectric motor, allowing the position of the transducer to be mechanically adjusted in X and Y directions (Fig. 1A). The transducer was connected to a water balloon filled with degassed water and coupled to the pig head through a water chamber. The pig’s head was supported and stabilized by a bite bar and two side-supports. We designed and manufactured the MRI-compatible frame (Fig. 1A) that combines all components on a single platform, which simplified the integration of the FUS system with the clinical MRI scanner (Ingenia 1.5 T, Philips Medical Systems, Inc., Cleveland, OH). A loop coil (dStream Flex L coil, Philips Medical Systems, Inc., Cleveland, OH) was placed on top of the FUS transducer for MR imaging (Fig. 1B, C). The FUS transducer was calibrated using a needle hydrophone in a water tank with a piece of the pig skull placed in front of the FUS transducer. The pressure levels reported in this study were based on these hydrophone measurements.

### Workflow for FUS-LBx in pigs

**Figure 2:**
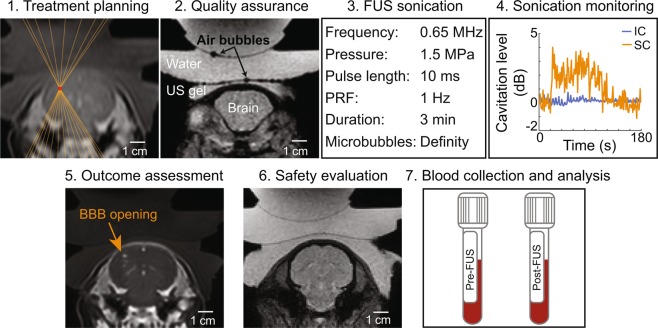
Workflow for FUS-LBx in pigs. The procedure started with treatment planning where the geometrical focus of the FUS transducer was aligned at the targeted brain location based on anatomic images acquired by T_1_-weighted and T_2_-weighted MRIs. Next, a T_2_^*^-weighted image, with the susceptibility artifact, was acquired to check whether the acoustic coupling media had air bubbles. If air bubbles were detected, as shown in the representative image, the preparation procedure was repeated until no bubbles were detected. FUS treatment began while monitoring cavitation activity with a PCD sensor. After treatment, contrast-enhanced T_1_-weighted imaging was performed to assess BBB opening via MRI contrast agent extravasation. Additionally, T_2_^*^-weighted imaging was used to detect hemorrhages. Blood samples were collected pre-FUS and post-FUS for the analysis of brain-specific biomarkers.

The overall workflow for FUS-LBx is summarized in Fig. 2. It consists of seven steps: treatment planning, quality assurance, FUS sonication, sonication monitoring, outcome assessment, safety evaluation, and blood sample analysis. Details of these steps are described below.

1. Treatment planning: A T_2_-weighted MRI scan was performed to image the pig head along with the FUS transducer (repetition time (TR): 1300 ms; echo time (TE): 130 ms; slice thickness: 1.2 mm; in-plane resolution: 0.58×0.58 mm;^2^ matrix size: 448×448; flip angle: 90°). The MRIs were imported to a software program (ThermoGuide, Image Guided Therapy, Pessac, France) to locate the geometrical focus of the transducer using 3-point triangulation. A specific brain location was targeted using a combination of mechanical movements by the MRI-compatible motor in 2D and electronic beam steering along the FUS axis.

2. Quality assurance: One key challenge in transcranial FUS energy delivery is ensuring sufficient acoustic coupling between the transducer and the animal skin. A T_2_^*^-weighted scan was obtained to visualize the presence of air bubbles in the acoustic coupling media (TR/TE: 710/23 ms; slice thickness: 2.5 mm; in-plane resolution: 0.98×0.98 mm;^2^ matrix size: 224×224; flip angle 18°). Susceptibility artifacts associated with T_2_^*^-weighted scans highlight the presence of air bubbles by amplifying the size of local magnetic field inhomogeneities, such as the air-liquid interface of bubbles in the coupling media. If air bubbles were detected, as shown in the representative image (Fig. 2), the preparation procedure was repeated until no bubbles were detected.

3. FUS sonication: Real-time PCD was performed to further verify sufficient acoustic coupling. If broadband emissions were present in the PCD when the FUS was turned on without microbubble injection, the most likely cause was due to air bubbles trapped in the coupling media that the T_2_^*^-weighted MRI scan was not able to detect. The experimental preparation procedure was repeated until there were no broadband emissions in the PCD. A T_1_-weighted MRI scan was acquired as a pre-treatment baseline for BBB permeability quantification (TR/TE: 5/2 ms; slice thickness: 1.5 mm; in-plane resolution: 0.68×0.68 mm;^2^ matrix size: 320×320; flip angle 10°). Then, FUS and PCD were turned on. The FUS parameters were: center frequency (f_0_): 0.65 MHz; pressure: 1.5 MPa; pulse repetition frequency: 1 Hz; pulse duration: 10 ms; treatment duration: 3 min. Fifteen seconds after FUS sonication began, perflutren lipid-shelled microbubbles (Definity, Lantheus Medical Imaging, North Billerica, MA) were administered intravenously with a dose of 0.2 mL/kg body weight and followed with a 3 mL saline flush. The dose of microbubbles (MB) was increased to 5× the recommended clinical dose to increase the harmonic emission while maintaining safe sonication^15^.

4. Sonication monitoring: PCD acquired at the beginning fifteen seconds before microbubble injection was used as the baseline for quantifying the signals acquired after microbubble injection. Referencing the method to calculate cavitation levels used by our previous publication^37^, a custom MATLAB script was written to process the acquired PCD data to evaluate the stable cavitation (SC) and inertial cavitation (IC) levels. Briefly, the stable and inertial cavitation levels were calculated as the root-mean-squared amplitudes of subharmonic (f_0_/2 ± 0.15 MHz) and broadband (0.3–2 MHz after removing f_0_/2 ± 0.15 MHz and nf_0_ ± 0.15 MHz where n = 1,2,3) signals, respectively. Statistical significance between pre-MB and post-MB cavitation doses was determined by the paired t-tests assuming Gaussian distribution.

5. Outcome assessment: Dynamic contrast-enhanced MRI (DCE-MRI) was performed to evaluate the dynamic extravasation of the MRI contrast agent, gadobenate dimeglumin (Gd-BOPTA; MultiHance, Bracco Diagnostics Inc., Monroe Township, NJ), from the blood circulation into brain tissue. Since Gd-BOPTA is too large to cross an intact BBB, the hyper-enhancement of signal in the T_1_-weighted images would indicate a successful BBB opening. One minute after starting the DCE-MRI scan, Gd-BOPTA was intravenously injected at a dose of 0.2 mL/kg and a rate of 2 mL/s. The DCE-MRI imaging sequence (TR/TE: 4.8/1.7 ms; slice thickness: 3 mm; in-plane resolution: 0.74×0.74 mm;^2^ matrix size: 336×336; flip angle: 25°; temporal resolution: 7.5 s) monitored the extravasation of Gd-BOPTA over 10 minutes. The quantitative analysis of contrast enhancement was performed with ROCKETSHIP^38^ using the extended Tofts model^39–41^ to estimate K^trans^, which is the influx rate constant for Gd-BOPTA to transfer from the blood to the tissue extravascular extracellular space and has been commonly used as an index of BBB permeability. The estimated K^trans^ values were averaged within a 5 voxel × 5 voxel region of interest at the FUS-targeted brain region (FUS + ) and the contralateral non-treated region (FUS-). Statistical significance between FUS + and FUS- was determined by the paired t-tests assuming Gaussian distribution.

Following the DCE-MRI sequence, a T_1_-weighted MRI scan was acquired (with the same parameters as the pre-treatment T_1_-weighted sequence) to further assess the BBB permeability. The outcome of FUS-induced BBB opening was evaluated by comparing the T_1_-weighted images of pre- and post-FUS using a custom MATLAB script. The analysis started by defining an elliptical ROI (major axis: 19 mm; minor axis: 8 mm) in the FUS + and FUS-. The ventricles were avoided in both ROIs because the hyperintensity of Gd-BOPTA in the ventricles would confound the calculation of hyper-enhancement in the tissue due to BBB disruption. Next, a voxel in the ROI was considered to contain an open BBB if the voxel intensity within the FUS + ROI was greater than 3× standard deviations above the mean intensity within the FUS- ROI. Then, the volume of BBB opening was estimated by calculating the sum of FUS + voxels for each image slice. Statistical significance between FUS + and FUS- groups was determined by the Wilcoxon matched-pairs signed-rank test. Additionally, the contrast-enhanced T_1_-weighted scan was overlaid with the planned transducer focus to guide the qualitative assessment of BBB opening and quantify the spatial offset between the planned and actual BBB opening.

6. *In vivo* safety assessment: The safety of the FUS-LBx technique was evaluated with a T_2_^*^-weighted MRI scan (with the same parameters as the pre-treatment T_2_^*^-weighted sequence) to detect FUS-induced hemorrhages approximately 1 hour after sonication. Hemorrhages would appear as hypointensity spots on the T_2_^*^-weighed images.

7. Blood collection and analysis: Blood was collected before and after FUS sonication to quantify the concentration of brain-specific biomarkers in the blood using enzyme-linked immunosorbent assays (ELISA). Because normal pigs were used, representative brain-specific biomarkers, GFAP and MBP, were selected for the blood analysis using the appropriate ELISA assay (Cusabio Biotech, Wuhan, China) and standard protocol provided by the manufacturer. Statistical significance between pre-FUS and post-FUS groups was determined by the paired t-test assuming Gaussian distribution.

### Histological analysis

After the FUS-LBx treatment was completed, the pigs were euthanized and tissues were collected. After the brain was fixed for 1 week in 10% formalin, the whole brain was placed in a 3D-printed brain slicing matrix to cut the brain into 3-mm thick slabs around the FUS treatment area. A gross examination of the target slice would determine the presence of FUS-induced macroscopic damage at the treatment site. The 3-mm thick slabs were embedded in paraffin and cut into 7 µm thin slices for hematoxylin and eosin (H&E) staining to examine red blood cell extravasation and cellular injury. The whole-brain horizontal slices were imaged on the Axio Scan.Z1 Slide Scanner (Zeiss, Oberkochen, Germany). A pathologist examined the stained slices and verified the results.

## Results

### FUS induced successful BBB opening

**Figure 3:**
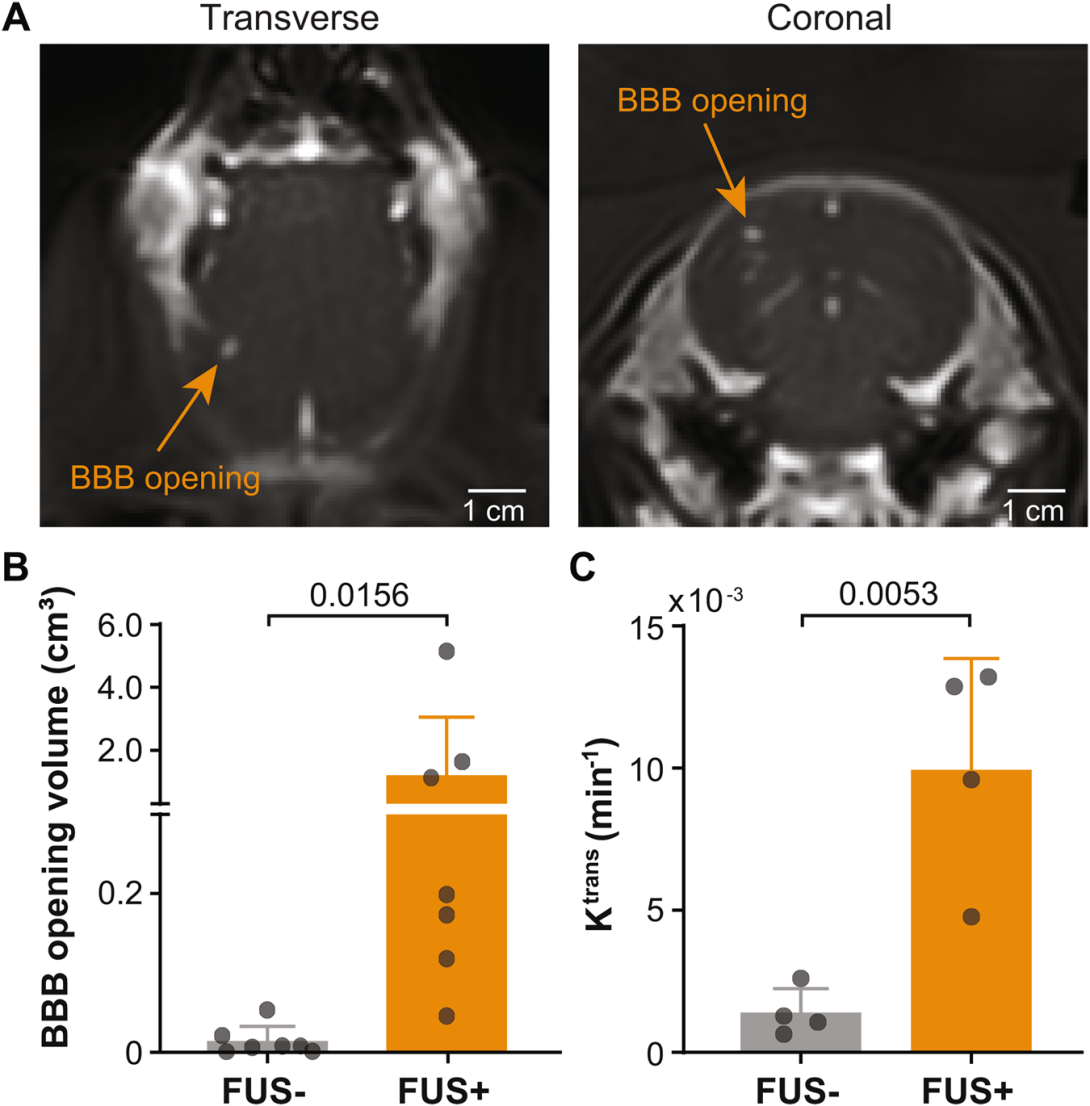
The customized MRgFUS system induced successful BBB opening in pigs. (**A**) Transverse and coronal T_1_-weighted MRIs of a pig show successful BBB opening as indicated by the MRI contrast agent extravasation at the FUS-targeted site. **(B)** Significant increase in BBB opening volume (p = 0.0156) at FUS-targeted brain region (FUS + ) and the contralateral non-treated region (FUS-). Each circular point represents the result obtained from each pig. **(C)** Significant increase in K^trans^ values (p = 0.0053) in the FUS + area compared with the FUS- site. K^trans^ estimation was performed for the last 4 pigs.

Successful BBB opening evidenced by contrast enhancement following FUS was achieved in 7 out of 8 pigs. One pig did not show obvious BBB opening, which could be attributed to the relatively large size of this pig (12.5 kg) compared to all other 7 pigs (8.16 ± 1.96 kg), leading to underestimated skull attenuation. Results obtained from the 7 pigs are presented in the following sections. Pharmacokinetic analysis of K^trans^ was conducted with 4 of the latest pigs. Figure 3A presents representative contrast-enhanced MRIs that show successful BBB disruption at the targeted brain location. The targeting accuracy as measured by the spatial offset between the target location and the actual BBB opening site was −1.9 ± 1.8 mm in the left-right direction (X), −0.4 ± 1.4 mm in head-foot direction (Y), and 5.3 ± 4.2 mm in the anterior-posterior direction (Z). The quantified BBB opening volume in the treated FUS + area (1.21 ± 1.84 cm^3^) was significantly greater (*p* = 0.0156) than the BBB opening volume (0.013 ± 0.018 cm^3^) in the contralateral FUS- site (Fig. 3B). The BBB permeability, quantified by K^trans^, of the targeted brain site (9.9 × 10^–3^ ± 3.9 × 10^–3^ min^−1^) was significantly greater (*p* = 0.0053) than that (1.4 × 10^–3^ ± 0.8 × 10^–3^ min^−1^) of the contralateral side (Fig. 3C).

### FUS enhanced the release of brain-specific biomarkers

**Figure 4:**
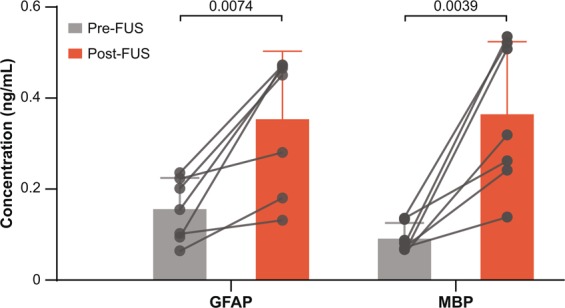
FUS enhanced the plasma concentration of GFAP and MBP. The concentrations of two brain-specific biomarkers, GFAP and MBP, significantly increased in blood collected post-FUS as compared with pre-FUS. Each circular point represents the result obtained from each pig.

FUS significantly enhanced the plasma concentration of the two brain-specific biomarkers, GFAP and MBP (Fig. 4). The GFAP concentration significantly increased (*p* = 0.0074) from 0.156 ± 0.068 ng/mL in pre-FUS blood samples to 0.353 ± 0.149 ng/mL in post-FUS blood samples. The MBP concentration in blood significantly increased (*p* = 0.0039) from 0.091 ± 0.034 ng/mL to 0.364 ± 0.159 ng/mL.

### No brain tissue damage was detected

**Figure 5:**
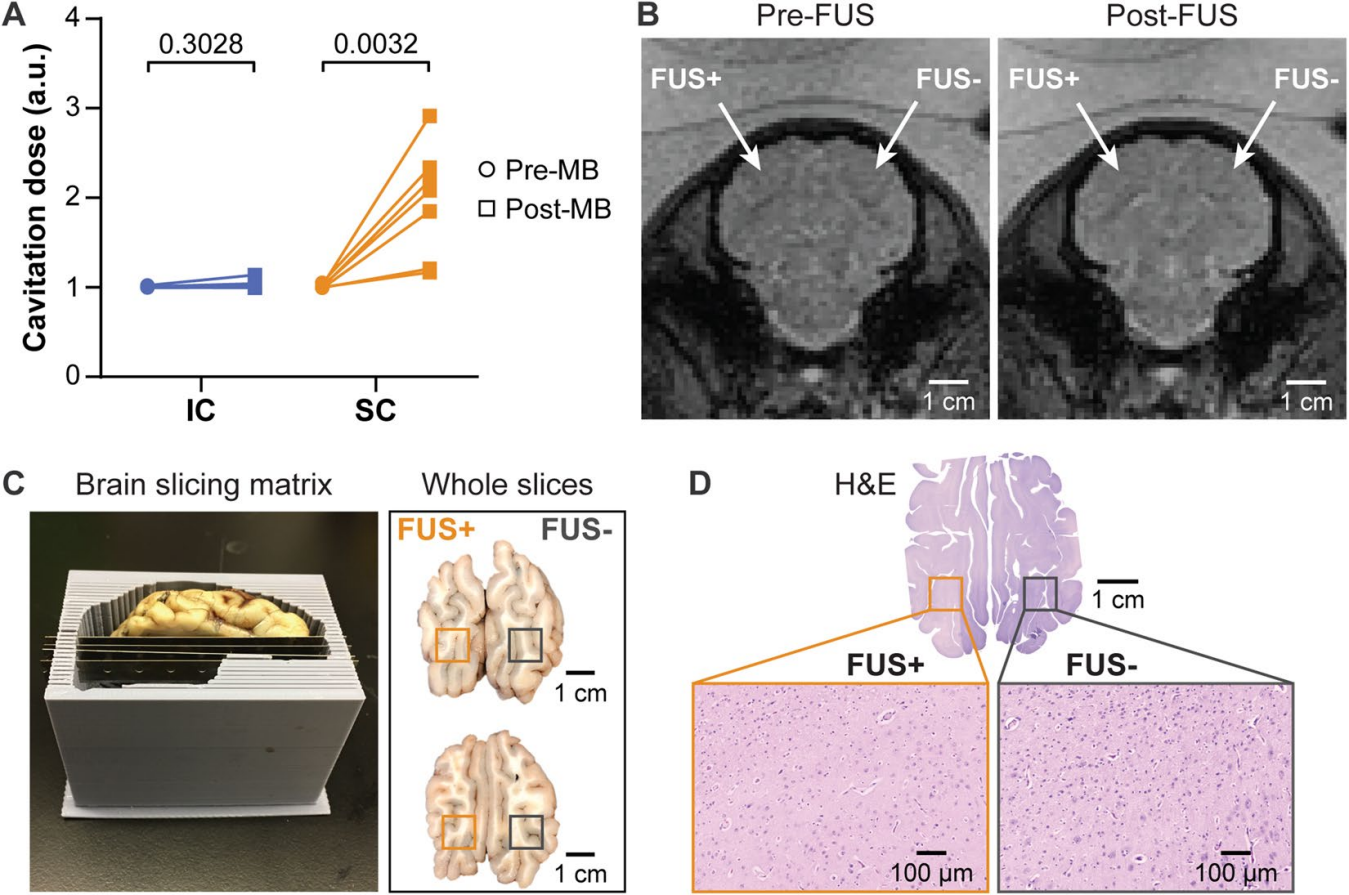
Safety assessment of FUS-LBx. **(A)** Calculated cavitation levels for each of the 7 pigs found no significant increase of IC after microbubble injection (post-MB) compared with before microbubble injection (pre-MB). **(B)** T_2_^*^-weighted images show no sign of hemorrhage after FUS treatment. **(C)** The 3D-printed brain slicing matrix was used to cut the brain into 3-mm thick slabs around the FUS treatment area. The gross pathological examinations did not find visible signs of tissue damage. The dark spots visible in the upper-right area of the whole brain slices were from uneven slicing where the lateral ventricle (black) was becoming visible on the right hemisphere but not visible on the left hemisphere. **(D)** H&E staining of the whole brain slices with microscopic views at FUS + and FUS- sites did not suggest cellular damage.

FUS cavitation monitoring, MRI, gross pathological assessment, and histological analysis did not detect any tissue damage. The lack of an IC increase post-MB compared to pre-MB suggests FUS did not induce violent MB activity that would lead to tissue damage (Fig. 5A). The representative T_2_^*^-weighted MRIs acquired at the same position as the T_1_-weighted MRIs shown in Fig. 3A, display no sign of hemorrhage in the treated site compared to the contralateral side (Fig. 5B). There were no macroscopic signs of bleeding or tissue damage on this representative brain surface or at the treatment site based on gross pathology (Fig. 5C). H&E staining of the target slices from a second representative subject did not find tissue damage or hemorrhage at the FUS-treated site (Fig. 5D).

## Discussion

Ever since FUS was introduced for brain applications in the 1940s, it has been used as a therapeutic tool^42,43^. Recently, FUS has been established as a promising tool for brain drug delivery by disrupting the BBB to enable the transfer of drugs from the blood circulation to the brain. The current study demonstrated the feasibility and safety of FUS to enhance the release of brain-specific biomarkers to the blood circulation in a porcine model for the diagnosis of brain diseases, suggesting that FUS is a promising theranostic tool for not only brain drug delivery but also brain disease diagnosis.

The noninvasive nature of the proposed FUS-LBx technique is especially advantageous over conventional neurosurgical tissue biopsies. FUS-LBx is proposed to complement tissue biopsies instead of replacing them, by enabling repeated longitudinal sampling to monitor treatment response, which is often impossible to perform by tissue biopsies for brain tumor patients. It may also provide complementary information in situations where assessment based on neuroimaging alone remains challenging, for example, to differentiate tumor pseudoprogression induced by treatment from the true tumor progression and recurrence as the treatment often interferes with tumor imaging properties^44^. FUS-LBx has the potential to radically advance the diagnosis, monitoring, and understanding of brain disease by precisely, rapidly, and safely identifying molecular signatures of brain diseases. Further, the technique presents a unique advantage in the assessment of spatially heterogeneous tumors. FUS can precisely target different locations of the tumor over time, thereby releasing biomarkers in a spatially-localized, temporally-resolved manner.

We were the first to propose that FUS-induced BBB opening enables safe two-way transfer between blood and brain. Our previous study validated this hypothesis using mouse models of glioblastoma^23^. The current study supports that this hypothesis is also valid in a large animal model. Our data showed that the customized MRgFUS device achieved successful BBB opening in the pig model as verified with the detection of MRI contrast agent extravasation from the blood circulation to the brain tissue and the significant increase in BBB permeability as measured by K^trans^ (Fig. 3). Meanwhile, the enhanced release of two brain-specific biomarkers from the brain to the blood circulation was confirmed by comparing the concentration of these biomarkers in the blood post-FUS with pre-FUS via ELISA quantification (Fig. 4). The enhanced biomarker release was achieved without causing detectable brain tissue damage (Fig. 5).

The application of FUS as a diagnostic tool provides a new pathway to clinical translation of the FUS technology. For drug delivery, FUS sonication needs to cover the whole targeted brain site (e.g., brain tumor) to distribute drugs throughout the tumor and kill every diseased cell. FUS sonication for LBx does not need to cover the entire tumor. Instead, FUS-LBx can pinpoint specific tumor locations for spatially targeted biomarker release. Therefore, FUS-LBx does not require the use of a complicated and expensive phase array, such as the INSIGHTEC Exablate Neuro system (costs > $2 M), which consists of 1,024 elements. Additionally, FUS-LBx does not need to combine FUS with neurotherapeutic drugs, thus simplifying FDA approval of the FUS technique.

The study is not without its limitations. First, one of the 8 pigs treated did not have a clear BBB opening, which could be attributed to the underestimation of skull attenuation. Future work will include PCD-based feedback control of FUS parameters to ensure the consistency of FUS sonication^45^. Second, this study investigated brain-specific biomarkers, not brain tumor-specific biomarkers. We verified that the enhanced release of these brain-specific biomarkers was induced by BBB disruption, not tissue damage. Regardless, future work is needed to demonstrate the application of FUS-LBx to detect disease-specific biomarkers by using a disease model. Third, only a single brain location was targeted and the enhancement in biomarker release was sufficient to establish the feasibility of the technique. However, future work is needed to determine the correlation between BBB opening volume and biomarker release efficiency to further optimize the FUS-LBx protocol. Fourth, various biomarkers have been investigated for cancer diagnosis and prognosis, such as circulating tumor DNAs, RNAs, and cancer-related proteins. Among them, circulating proteins are often larger than other biomarkers, which may be associated with lower release efficiency. Future studies are needed to explore the application of FUS in the release of other biomarkers and evaluate the biomarker type-dependency of FUS-LBx.

## Conclusions

This study demonstrated the feasibility and safety of FUS-mediated release of brain-specific biomarkers from the brain to the blood in a pig model. A customized MRgFUS system was designed and built for BBB disruption in pigs, and workflow for FUS-LBx in the pig model was established. Localized BBB opening was verified with contrast-enhanced MRI, while the increased concentration of brain-specific biomarkers, GFAP and MBP, in blood was confirmed with ELISA quantification. This study was the first to verify the hypothesis that FUS-induced BBB opening enables two-way transfer across the BBB in a large animal model. This proof-of-concept study in a large animal model laid the foundation for the future clinical translation of FUS-LBx as a noninvasive and localized brain tumor liquid biopsy technique. This study suggests that FUS is a promising theranostic tool for not only brain drug delivery but also brain disease diagnosis in combination with liquid biopsies.
